# Effect of Butoconazole Nitrate 2% Vaginal Cream and Miconazole Nitrate 2% Vaginal Cream Treatments in Patients with Vulvovaginal Candidiasis

**DOI:** 10.1155/S1064744996000658

**Published:** 1996

**Authors:** Myra A. Lappin, Doris C. Brooker, Carol A. Francisco, Joan Dorfman

**Affiliations:** 1 Student Health Service San Francisco State University MS 4200 1600 Holloway Avenue San Francisco CA 94132-4200 USA; 2 Department of Obstetrics and Gynecology University of Minnesota Minneapolis MN USA; 3 Department of Biostatistics Syntex Laboratories, Inc. Palo Alto CA USA; 4 Department of Clinical Investigations Syntex Laboratories, Inc. Palo Alto CA USA

## Abstract

In a multicenter, randomized, invesgtigator-blind, parallel study, 398 patients were dispensed topical butoconazole nitrate 2% cream for 3 days (n = 199) or miconazole nitrate 2% cream for 7 days (n = 199) for vaginal use. Efficacy analyses included 254 patients with culture-confirmed *Candida* (119 butoconazole and 135 miconazole users). Of the 398 patients issued study medication, 9 were lost to follow-up. Therefore, safety analyses included 389 patients (197 butoconazole and 192 miconazole users). Evaluations upon admission and approximately 8 and 30 days post-treatment included *Candida* cultures, potassium hydroxide (KOH) wet mounts, and vulvovaginal examinations, with rating of vulvovaginal signs and symptoms using a 4-point scale. Rates of clinical cure (based on sign/symptom scores), microbiologic cure (based on cultures and wet mounts), and therapeutic cure (both clinical and microbiologic cures) were assessed and were to be similar between the regimens. Therapeutic cure rates were 57.8% and 61.4% for butoconazole and miconazole, respectively. Three-day butoconazole treatment was as safe and effective as 7-day miconazole therapy in treating vulvovaginal candidiasis.


Infectious Diseases in Obstetrics and Gynecology 4:323–328 (1996)
© 1997 Wiley-Liss, Inc.

### Effect of Butoconazole Nitrate 2% Vaginal Cream and Miconazole Nitrate 2% Vaginal Cream Treatments in Patients with Vulvovaginal Candidiasis

Myra A. Lappin, [1]* Doris C. Brooker,[2] Carol A. Francisco,[3] and Joan Dorfman[4]

[1]*Student Health Service, San Francisco State University, San Francisco, CA*
[2]*Department of Obstetrics and Gynecology, University of Minnesota, Minneapolis, MN*
[3]*Department of Biostatistics, Syntex Laboratories, Inc., Palo Alto, CA*
[4]*Department of Clinical Investigations, Syntex Laboratories, Inc., Palo Alto, CA*

ABSTRACT

In a multicenter, randomized, investigator-blind, parallel study, 398 patients were dispensed topical butoconazole nitrate 2% cream for 3 days (n = 199) or miconazole nitrate 2% cream for 7 days (n = 199) for vaginal use. Efficacy analyses included 254 patients with culture-confirmed *Candida* (119 butoconazole and 135 miconazole users). Of the 398 patients issued study medication, 9 were lost to follow-up. Therefore, safety analyses included 389 patients (197 butoconazole and 192 miconazole users). Evaluations upon admission and approximately 8 and 30 days post-treatment included *Candida* cultures, potassium hydroxide (KOH) wet mounts, and vulvovaginal examinations, with rating of vulvovaginal signs and symptoms using a 4-point scale. Rates of clinical cure (based on sign/symptom scores), microbiologic cure (based on cultures and wet mounts), and therapeutic cure (both clinical and microbiologic cures) were assessed and were to be similar between the regimens. Therapeutic cure rates were 57.8% and 61.4% for butoconazole and miconazole, respectively. Three-day butoconazole treatment was as safe and effective as 7-day miconazole therapy in treating vulvovaginal candidiasis. Infect. Dis. Obstet. Gynecol. 4:323–328, 1996. © 1997 Wiley-Liss, Inc.

KEY WORDS

antifungal; *Candida;* yeast; cure; clinical; butoconazole; miconazole

Butoconazole nitrate 2% cream and miconazole nitrate 2% cream have both been used as effective intravaginal antifungal agents to treat vulvovaginal candidiasis, one of the most common gynecological problems.[1,2]

It is important for women who are first-time sufferers to consult their physicians before using any over-the-counter products to treat vulvovaginal candidiasis, thus ensuring an accurate diagnosis. With the diagnosis of vulvovaginal candidiasis comes the challenge to health care professionals of patient compliance. Several factors may influence compliance, including length of antifungal therapy. To that end, physician recommendations which

The following practitioners also participated in this study: Dale Brown, Jr., Houston, TX; Fred W. Schnepper, Chula Vista, CA; Kathleen O'Hare, Champaign, IL; Stephen F. Gordon, Atlanta, GA; and the Butoconazole OTC Study Group, including Robert I. Fulmer, Austin, TX; Russel Graham, Altamonte Springs, FL; Judson E. Jones, Aurora, IL; Joseph Liotti, West Orange, NJ; Elizabeth Nye, Chicago, IL; Anthony Puopolo, Milford, MA; Terry O'Reilly, Olympia, WA; Meenakshi Patel, Dayton, OH; William Schultz, Springfield, IL; John M. Stafford, St. Joseph, MN; Kumjad Unnoppet, Birmingham, AL; Arthur Waldbaum, Denver, CO; Paul F. Whitsitt, Oshawa, Ontario, Canada, Marian Diermayer, Co-investigator, SHS, SFSh, San Francisco, CA.

*Correspondence to: Myra A. Lappin, MD, Student Health Service, San Francisco State University, MS 4200, 1600 Holloway Avenue, San Francisco, CA 94132-4200.

Received 18 July 1996

Accepted 14 January 1997

encourage compliance may include short-term antifungal therapy.[3,4]

The primary objective of this study was to compare the effectiveness and safety of 3-day butoconazole with 7-day miconazole treatments in women with vulvovaginal candidiasis. Therapeutic, microbiologic, and clinical cure rates were determined after consecutive daily intravaginal treatment with either butoconazole nitrate 2% cream (3 days) or miconazole nitrate 2% cream (7 days).

### SUBJECTS AND METHODS

The study had a multicenter, randomized, investigator-blind, parallel design. Eligible patients were randomly assigned to treatment once daily at bedtime with either butoconazole nitrate cream for 3 consecutive days or miconazole nitrate cream for 7 consecutive days. Butoconazole nitrate was presented to patients in one box containing 3 applicators prefilled with butoconazole nitrate cream. Miconazole nitrate was presented to patients in one box containing 7 applicators and a tube of miconazole cream. Boxes were identically packaged.

### Admission Criteria

Patients admitted to the trial were generally healthy women with pseudohyphae on potassium hydroxide (KOH) wet mount, which was confirmed by a positive *Candida* culture. Enrolled patients signed an informed consent and agreed to abstain from sexual intercourse during the treatment period and to use condoms for the remainder of the study period. During the study they also agreed to abstain from using any vaginal product containing nonoxynol-9, vaginal douches, other vaginal or vulvar topical therapeutics, vaginal contraceptives, or feminine sprays. Excluded from the study were patients with evidence of trichomonads or clue cells (indicative of bacterial vaginosis); vulvar, cervical, or vaginal lesions other than candidiasis (e.g., herpes); diabetes mellitus, acquired immunodeficiency syndrome (AIDS), or any uncontrolled systemic disease; and urinary tract infection or any genitourinary condition including sexually transmitted disease. Candidates were also excluded if they were pregnant or breast-feeding; had been treated for vulvovaginal candidiasis within the week preceding study entry; had used any vaginal preparations within 24 h before study entry; were receiving anti-inflammatory, antihistamine, or analgesic drugs on an ongoing basis; or would require oral antibiotics during the study treatment.

### Assessments

Patients had an admission visit and two follow-up visits scheduled as close as possible to 8 and 30 days after the end of treatment. At the admission visit, a medical history was obtained and a physical including vulvovaginal examination was performed. Vulvovaginal signs (erythema, swelling, excoriation, ulceration) and symptoms (burning, itching) were recorded and rated on a 4-point scale (0 = none; 1 = mild; 2 = moderate; 3 = severe). Each patient had a sodium chloride and KOH wet mount, *Candida* yeast culture (including species identification) to confirm the presence of *Candida*, Pap smear (if not done within the previous 6 months), urine pregnancy test (if the patient was of child-bearing potential), and urinalysis. At the two follow-up visits (visits 2 and 3), KOH wet mount and vaginal *Candida* culture were obtained. In addition, vulvovaginal signs and symptoms, concomitant medication use, and adverse events were monitored.

### Cure Definitions

Clinical response was assessed at both follow-up visits on the basis of presence or absence of vulvovaginal signs/symptoms (e.g., burning, itching, erythema, swelling, excoriation, and ulceration) rated on a 4-point categorical scale (i.e., 0 = none; 1 = mild; 2 = moderate; 3 = severe). *Clinical cure* was achieved when all of the following conditions were met at a follow-up visit: all vulvovaginal sign and symptom severity scores were 0 or 1; each of the patient's sign and symptom scores was not greater (getting worse) than the score for the previous visit (baseline or the first follow-up visit); and at least half of the sign and symptom scores that were ≥1 in the preceding visit (including baseline) improved to scores of 0 or 1. There was one exception to these guidelines: a patient was considered clinically cured at the second follow-up visit if all assessments at the first follow-up visit were 0 except for one score of 1, and the assessments remained the same at the second follow-up visit (with no missing assessments at either visit). Patients not clinically cured were considered clinical treatment failures. Any patient who was not clinically cured at the first follow-up visit was excluded from the clinical cure rate analysis at the second follow-up visit.

Assessment of microbiologic response was based on results of fungal culture and KOH wet mount. A patient was considered *microbiologically cured* if her fungal culture was negative and her KOH wet mount result was either negative or missing. Any patient who was not microbiologically cured at the first follow-up visit was excluded from the microbiological cure rate analysis at the second follow-up visit.

*Therapeutic cure* was defined as clinical and microbiologic cures at both follow-up visits, and therapeutic failure as either a clinical or a microbiologic failure at either follow-up visit. Patients who were never clinical or microbiologic failures, but who did not have all four cure rate results were considered to have insufficient data for determination of their results. Therefore, their results were considered missing, and they were excluded from the therapeutic cure analysis, as well as from the clinical or microbiologic cure analysis for that visit.

### Statistical Analysis

Baseline comparability of the treatment groups was assessed using a two-way analysis of variance (ANOVA) with factors of treatment, investigator, and treatment-by-investigator interaction for continuous variables and a Cochran-Mantel-Haenszel test (controlling for investigator) for discrete variables. A significance level of 0.10 was used for baseline treatment comparisons and for testing treatment-by-investigator interaction.

The primary efficacy variables were the clinical and microbiologic cure rates at each follow-up visit and overall therapeutic cure rate. Valid follow-up visits had to be within a 7 day to 15 post-treatment day window for visit 2 and within a 27 day to 45 post-treatment day window for visit 3. Clinical and microbiologic cure rates for each treatment group were calculated at each follow-up visit by dividing the number of cured patients by the total number of patients analyzed. As an alternative, a more conservative procedure for the second follow-up visit was also applied. This entailed counting patients as failures if they were cured at the first follow-up visit (visit 2) but had no results available from the last visit (visit 3). Therapeutic cure rate was calculated by dividing the number of therapeutic cures by the total number of patients analyzed, excluding those who had insufficient data for this analysis.

Cure rates were analyzed as two independent binomial proportions. Both a 95% two-tailed confidence interval and a lower limits of 95% one-tailed confidence interval for the difference in cure rates (butoconazole minus miconazole) were calculated using a normal approximation to the binomial distribution. Yate's continuity correction was incorporated.[5] Cure rates for the two treatment groups were also compared using the Cochran-Mantel-Haenszel test for general association, controlling for investigator,[6] and a z-test that used the minimum-variance estimator of the overall cure rate differences across sites (butoconazole minus miconazole) in order to test the hypothesis that the treatment difference was zero.[7]

The use of these statistical analyses was justified by the assumption that there was no treatment-by-investigator interaction (i.e., that data from all sites could be pooled). This assumption was tested by establishing the homogeneity of the treatment odds ratios (butoconazole odds divided by miconazole odds) across investigator sites using Zelen's[8] test and then testing that the common odds ratio was equal to one,[9] and by a chi-square statistic that examined the homogeneity of cure rate differences across the sites.[7,10] Adverse events were recorded and summarized using the COSTART mapping system.[11]

### RESULTS

Nineteen investigators enrolled 403 patients, 5 of whom did not receive the study medication after enrollment, were not randomized, and were therefore excluded from all analyses. Thus, 398 patients (199 in each treatment group) were randomly assigned to treatment with butoconazole nitrate cream or miconazole nitrate cream. Nine patients (2 butoconazole and 7 miconazole users) were lost to follow-up and were excluded from the safety analyses, which included 389 patients (197 butoconazole and 192 miconazole users). Included in the efficacy analyses were 254 participants, comprising 119 butoconazole recipients and 135 miconazole users. Exclusions from the efficacy analyses are detailed in Table 1.

The treatment groups in the efficacy analyses were comparable with respect to demographic characteristics of age (mean 32 years, SD 10 years; range 17–67 years), ethnicity (73% European, 15% African American, 8% Latina, 3% Asian-American, 1% other), and height (mean 64.5 in., SD 2.8 in.;

**TABLE 1. Patients excluded from efficacy analyses**

| Reason for exclusion | Total (%) | Butoconazole (%) | Miconazole (%) |
|---|---|---|---|
| No confirmation that study drug was used | 9(2) | 2(1) | 7(4) |
| Negative or missing fungal culture/KOH* | 92(23) | 53(27) | 39(20) |
| Positive urine glucose or dysplasia on Pap smear | 4(1) | 4(2) | 0 |
| Non-compliance with study medication | 14(3) | 7(4) | 7(4) |
| Medication applied but no follow-up visits | 2(<1) | 2(1) | 0 |
| First follow-up visit outside 7–15 day window | 13(3) | 7(4) | 6(3) |
| Used prohibited concomitant medication | 2(<1) | 1(1) | 1(1) |
| Evidence of trichomonads, *Gardnerella*, bacterial vaginitis, or cervicitis | 8(2) | 4(2) | 4(2) |
| Total excluded** | 144(36) | 80(40) | 64(32) |
| Total included | 254(64) | 119(60) | 135(68) |
| Total enrolled, received study drug | 398 | 199 | 199 |

*$P$ = 0.071 (Cochran-Mantel-Haenszel test).
**$P$ = 0.079 (Cochran-Mantel-Haenszel test).

range 51–72 in.). There were significant differences between the treatment groups in systolic blood pressure ($P$ = 0.06), and there were significant treatment-by-investigator interactions for weight ($P$ = 0.003), systolic blood pressure ($P$ = 0.098), and diastolic blood pressure ($P$ = 0.053). These variables were not considered to have an impact on the cure rate outcomes. The groups were comparable in baseline (pretreatment) signs of erythema, ulceration, and presence and quantity of discharge. There were significant differences between the groups in baseline vulvovaginal burning ($P$ = 0.004) and itching ($P$ = 0.078), with a greater proportion in the butoconazole group having severe symptoms (burning: 19 patients [16%] for butoconazole and 8 patients [6%] for miconazole; itching: 34 patients [29%] for butoconazole and 26 patients [19%] for miconazole). Similarly, statistically significant differences were also found for swelling and excoriation at baseline ($P$ = 0.04 and 0.077, respectively), and these significant differences in baseline characteristics were due to a treatment-by-investigator interaction, with most cases of swelling or excoriation reported by only a few investigators. When the Cochran-Mantel-Haenszel test was applied to the data collapsing on investigator, there were no statistically significant differences ($P \geq$ 0.197).

### Efficacy

Cure rate data were pooled across all investigators because the results of tests for homogeneity among investigators showed no statistically significant treatment-by-investigator interactions (Zelen's test $P$ values = 0.152–0.916; common odds ratios not significantly different from 1 and all 95% confidence intervals for these ratios contained the value 1; and chi-square $P$ values = 0.152–0.514).

Clinical, microbiologic, and therapeutic cure rates for butoconazole and miconazole were similar between treatment groups, regardless of whether patients with missing/insufficient data from the second follow-up examination were excluded from the analysis or included as treatment failures (Table 2). No differences between butoconazole nitrate and miconazole nitrate were statistically significant. Clinical cure rates at the first follow-up examination (approximately 8 days post-treatment) were 92.4% for butoconazole and 94.8% for miconazole; at the second follow-up assessment (approximately 30 days post-treatment), clinical cure rates were 88.5% for butoconazole and 85.7% for miconazole. Microbiologic cure rates at the first follow-up visit were 88.1% for butoconazole and 89.6% for miconazole, and at the second follow-up examination they were 75.5% and 78.4%, respectively. An alternate procedure counted patients as failures who were cured at the first follow-up visit but missing their second follow-up data. This alternate, more conservative procedure for estimating clinical and microbiologic cure rates resulted in somewhat lower clinical cure rates at the second follow-up visit (butoconazole: 77.3%; miconazole: 79.7%) and microbiologic cure rates (butoconazole: 68.3%; miconazole: 72.5%). Therapeutic cure rates were 57.8% for butoconazole and 61.4% for miconazole.

Eighteen patients, 11 in the butoconazole group and 7 in the miconazole group, had fungal cultures positive for organisms other than *C. albicans*. In the

TABLE 2. Summary of cure rates[a]

| Cure rate | Treatment groups: Butoconazole | Miconazole | Difference[b] (%) | Two-sided 95% confidence interval (%) |
|---|---|---|---|---|
| First follow-up | | | | |
| Clinical | 110/119 (92.4%) | 128/135 (94.8%) | −2.4 | −9.2, 4.5 |
| Microbiologic | 104/118 (88.1%) | 120/134 (89.6%) | −1.4 | −10.0, 7.2 |
| Second follow-up | | | | |
| Clinical | 85/96 (88.5%) | 102/119 (85.7%) | 2.8 | −7.1, 12.7 |
| Microbiologic | 71/94 (75.5%) | 87/111 (78.4%) | −2.8 | −15.4, 9.7 |
| Therapeutic cure rate | 63/109 (57.8%) | 78/127 (61.4%) | −3.6 | −17, 9.8 |

[a]Patients with missing/insufficient data from the second follow-up visit were excluded from the analysis for that visit.
[b]Butoconazole cure rate minus miconazole cure rate.

butoconazole group, 6 patients had *Torulopsis glabrata*, 2 had *C. tropicalis*, 2 *C. parapsilosis*, and 1 other non-*albicans Candida*. In the miconazole group, 4 had *T. glabrata*, 1 *C. stellatoidea*, 1 *C. parapsilosis*, and 1 *C. tropicalis*. Therapeutic cures for butoconazole recipients were achieved in 4 of the 9 patients valid for efficacy analysis, 2 with *C. parapsilosis*, 1 with *C. tropicalis*, and 1 with *T. glabrata*. A therapeutic cure was achieved in 1 of the 5 miconazole users valid for efficacy analysis. This subject had *C. tropicalis*. Four patients with fungal cultures positive for non-*albicans* organisms were not analyzed (i.e., 2 treated with butoconazole nitrate and 2 with miconazole nitrate).

### Safety

There were no deaths and no serious adverse events reported. Three butoconazole-treated patients withdrew prematurely from the study because of non-serious adverse events: increased burning and itching; vaginal burning; and itching and rash. One miconazole user withdrew prematurely because of moderate lower abdominal cramping. All of these events were considered probably or possibly related to study medication. Two patients withdrew prematurely because of laboratory abnormalities (suspicious Pap smear with dysplasia) and 5 because of intercurrent illnesses (vaginal trichomoniasis, bladder pressure, cervicitis, bacterial vaginitis, streptococcus group B infection); these events were considered by the investigators to be probably not related to the study medications.

Of all 389 patients evaluated in the safety analysis, 148 (38%) reported a total of 287 adverse events: 70 of 197 butoconazole recipients (36%) and 78 of 192 miconazole users (41%). Overall for both treatment arms, most adverse events (88%) were rated as mild or moderate in severity and 67% of all events were considered to be probably not related to butoconazole nitrate or miconazole nitrate cream. The most commonly reported adverse event was "headache" (butoconazole nitrate group: 9%; miconazole nitrate group: 9%).

Events related to the urogenital system were reported by 60 patients (15%): 29 (15%) in the butoconazole group and 31 (16%) in the miconazole group. Urogenital complaints reported by more than 3 patients were pain, pruritus, leukorrhea, and vaginitis, as detailed in Table 3. Of the adverse events considered by the investigators to be possibly or probably related to study medications, among the most frequently reported were vaginal pain or itching, both of which were also symptoms of the underlying disease.

### DISCUSSION

The lack of statistically significant differences between the 3-day butoconazole regimen and the 7-day miconazole regimen with respect to microbiologic, clinical, or therapeutic cure rates coupled with large sample sizes indicate that the two study medications were equally effective in the treatment of vulvovaginal candidiasis. Although the study groups were demographically well matched, clinical and therapeutic cure rate differences in this

TABLE 3. Urogenital adverse events reported by >3 patients

| | No. of patients reporting | | |
|---|---|---|---|
| Event | Butoconazole | Miconazole | Total |
| Pain | 15 | 13 | 28 |
| Pruritus | 12 | 9 | 21 |
| Leukorrhea | 1 | 6 | 7 |
| Vaginitis | 3 | 2 | 5 |

study may have been affected by the preponderance of patients with severe itching or burning in the butoconazole group (16% and 29%, respectively, vs. 6% and 19%, respectively, for miconazole). Nonetheless, the study showed that both drugs were equally effective in alleviating vaginitis symptoms at visit 2, with continued or maintained recovery at visit 3. Both study drugs were well tolerated, and no serious adverse events were reported.

The findings of this study support those of Kaufman et al.,[12] whose large-scale study also had a randomized, investigator-blind design, and demonstrated that 3-day butoconazole treatment was equal in efficacy to 7-day miconazole therapy. These authors speculated that the 3-day regimen could have an advantage over the 7-day regimen in that patients might be more likely to comply with the full therapeutic regimen if given a shorter treatment schedule.

Although the majority of patients in the present study had fungal cultures positive for *C. albicans*, 18 patients had fungal cultures positive for organisms other than *C. albicans*. Of the patients with non-*albicans* species, therapeutic cures were achieved in 4 of 9 butoconazole users and 1 of 5 miconazole users. Two butoconazole patients were excluded and 2 miconazole patients were excluded from analysis. The sample in this subgroup was too small to draw statistically significant or clinically relevant conclusions. In a separate in vitro study, however, butoconazole was found to be more effective at lower concentrations than miconazole and other azoles against non-*albicans* species.[2] In tests of 200 vaginal yeast isolates of 9 species, including 100 isolates of non-*albicans* species, Lynch and Sobel[2] found only 2 isolates of *C. kansei* in which butoconazole >0.001 μg/ml was required to reduce microorganism growth by 80%. The minimal inhibitory concentration of miconazole was 10-fold higher than the butoconazole concentration in 173 isolates of *C. albicans*, *C. glabrata*, and *C. parapsilosis*. Still higher concentrations of other azoles were required for equivalent inhibitory effect.

### CONCLUSIONS

Butoconazole nitrate 2% cream applied intravaginally for 3 days and miconazole nitrate 2% cream applied for 7 days demonstrated comparable efficacy in the treatment of vulvovaginal candidiasis. In addition, both treatments were well tolerated and were not associated with any serious adverse events. These results indicate that butoconazole nitrate 2% cream, applied once daily for 3 days, is comparable to an approved over-the-counter product.

### ACKNOWLEDGMENTS

This study was funded in part by a grant from Syntex Laboratories, Inc.

### REFERENCES

1. Adamson DG: Three-day treatment of vulvovaginal candidiasis. Am J Obstet Gynecol 158:1002–1005, 1988.
2. Lynch ME, Sobel JD: Comparative in vitro activity of antimycotic agents against pathogenic vaginal yeast isolates. J Med Vet Mycol 32:267–274, 1994.
3. Nixon SA: Vulvovaginitis: The role of patient compliance in treatment success. Am J Obstet Gynecol 165: 1207–1209, 1991.
4. Hunt IM, Jordan B, Irwin S, Browner CH: Compliance and the patient's perspective: Controlling symptoms in everyday life. Cult Med Psychiatry 13:315–334, 1989.
5. Fleiss JL: Statistical Methods for Rates and Proportions. 2nd ed. New York: John Wiley & Sons, 1981.
6. Kuritz SJ, Landis JR, Koch GG: A general overview of Mantel-Haenszel methods: Applications and recent developments. Annu Rev Public Health 9:123–160, 1988.
7. Rao CR: Linear Statistical Inference and Its Applications. 2nd ed. New York: John Wiley & Sons, 1973.
8. Zelen M: The analysis of several 2 × 2 contingency tables. Biometrika 58:129–137, 1971.
9. Gart J: Point and interval estimation of the common odds ratio in the combination of 2 × 2 tables with fixed marginals. Biometrika 57:471–475, 1970.
10. Uesaka H: Test for interaction between treatment and stratum with ordinal responses. Biometrics 49:123–129, 1993.
11. U.S. Food and Drug Administration: "COSTART": Coding Symbols for Thesaurus of Adverse Reaction Terms. Publication NTIS PD 90-114026 AS. 3rd ed. Rockville, MD: Center for Drugs and Biologics, Division of Drug and Biological Products Experience, 1989.
12. Kaufman RH, Henzl MR, Brown D Jr, et al: Comparison of three-day butoconazole treatment with seven-day miconazole treatment for vulvovaginal candidiasis. J Reprod Med 34:479–483, 1989.

## References

[PDF_00519] Adamson G. D. (1988). Three-day treatment of vulvovaginal candidiasis.. Am J Obstet Gynecol.

[PDF_00527] Hunt L. M., Jordan B., Irwin S., Browner C. H. (1989). Compliance and the patient's perspective: controlling symptoms in everyday life.. Cult Med Psychiatry.

[PDF_00550] Kaufman R. H., Henzl M. R., Brown D., Horner D. S., Krauss R. H., Mehlisch D. R., Moore D. E., Prentice R. L. (1989). Comparison of three-day butoconazole treatment with seven-day miconazole treatment for vulvovaginal candidiasis.. J Reprod Med.

[PDF_00532] Kuritz S. J., Landis J. R., Koch G. G. (1988). A general overview of Mantel-Haenszel methods: applications and recent developments.. Annu Rev Public Health.

[PDF_00521] Lynch M. E., Sobel J. D. (1994). Comparative in vitro activity of antimycotic agents against pathogenic vaginal yeast isolates.. J Med Vet Mycol.

[PDF_00524] Nixon S. A. (1991). Vulvovaginitis: the role of patient compliance in treatment success.. Am J Obstet Gynecol.

[PDF_00542] Uesaka H. (1993). Test for interaction between treatment and stratum with ordinal responses.. Biometrics.

